# Supplementary material for: Altered projection-specific synaptic remodeling and its modification by oxytocin in an idiopathic autism marmoset model
Source: Commun Biol. 2024 May 27;7:642. doi: 10.1038/s42003-024-06345-9 (PMC11130163; doi:10.1038/s42003-024-06345-9)
Supplement: Supplementary file 2 — Supplementary Information [file 42003_2024_6345_MOESM2_ESM.pdf]

Supplementary Information

Altered projection-specific synaptic remodeling and its modification by oxytocin in an idiopathic autism marmoset model

Jun Noguchi, Satoshi Watanabe, Tomofumi Oga, Risa Isoda, Keiko Nakagaki, Kazuhisa Sakai, Kayo Sumida, Kohei Hoshino, Koichi Saito, Izuru Miyawaki, Eriko Sugano, Hiroshi Tomita, Hiroaki Mizukami, Akiya Watakabe, Tetsuo Yamamori & Noritaka Ichinohe

This PDF file includes:

Supplementary Figure 1 to 12

Legends for Supplementary Figures

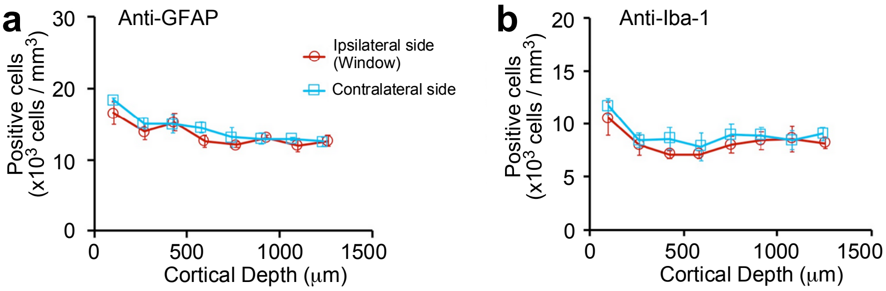

Supplementary Figure 1 The distributions of glial cell density along the cortical depth were not significantly different between the two-photon observation window side (ipsilateral) hemisphere and the opposite (contralateral) hemisphere. **a** Activated astrocyte marker GFAP postive cell density was calculated. Distinct sign of astrocyte activation under the imaging window was not detected ($P = 0.26$ at 83-115 µm cortical depth; Mann-Whitney U-test; Total 12 sites on the dorsomedial prefrontal cortex ($1.1x10^{-3}$ mm^3 for one site) from three marmosets). **b** Microglia marker Iba-1 postive cell density was calculated as in (**a**). Obvious sign of glial cell activation under the imaging window was not detected. ($P = 0.27$ at 92-112 µm cortical depth; Mann-Whitney U-test; Total 12 sites ($1.1x10^{-3}$ mm^3 for one site) from three marmosets). Data are represented as mean ± s.e.m.

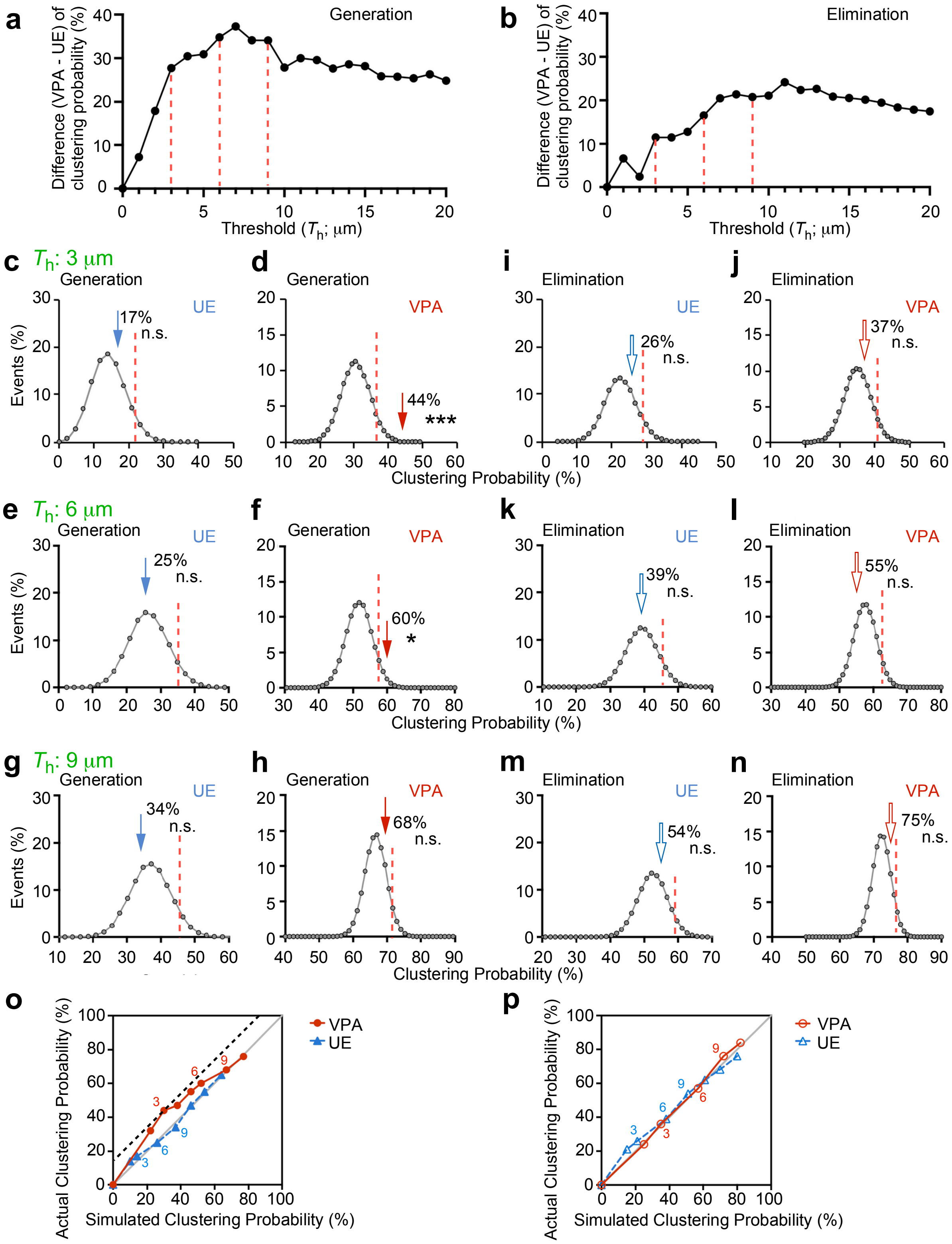

a
Generation
Difference (VPA - UE) of clustering probability (%)
Threshold (T_h; μm)
b
Elimination
c T_h: 3 μm
d
e T_h: 6 μm
f
g T_h: 9 μm
h
i
j
k
l
m
n
Generation
Elimination
UE
VPA
Events (%)
Clustering Probability (%)
17% n.s.
44% ***
26% n.s.
37% n.s.
25% n.s.
60% *
39% n.s.
55% n.s.
34% n.s.
68% n.s.
54% n.s.
75% n.s.
o
p
VPA
UE
Actual Clustering Probability (%)
Simulated Clustering Probability (%)

Supplementary Figure 2 Analysis of clustering probability for different clustering threshold values. a, b The relationship between the clustering thresholds (T_h) and differences between the VPA-exposed and UE animals in clustering probabilities of newly generated (**a**) or eliminated (**b**) spines. The threshold values applied to the subsequent analyses were shown by red broken lines. See **Materials and Methods** for details. **c–h** Validation of clustered spine generation by Monte Carlo simulation. Circles connected with gray lines represent probability plots of clustering events from 100,000 simulations; the actual numbers of spine clusters are represented by arrows for T_h = 3 μm (**c**, **d**; *P* = 0.24 and 0.00042; *n* = 44 and 86 newly generated spines in 14 and 12 dendrites in UE and VPA-exposed animals, respectively) and for T_h = 6 μm (**e**, **f**; *P* = 0.53 and 0.016), and for T_h = 9 μm (**g**, **h**; *P* = 0.63 and 0.29). **i–n** Validation of clustered spine elimination as in (**c**–**h**). The actual numbers of spine clusters are represented by arrows for T_h = 3 μm (**i**, **j**; *P* = 0.20 and 0.26; *n* = 68 and 100 newly eliminated spines in UE and VPA-exposed animals, respectively) and for T_h = 6 μm (**k**, **l**; *P* = 0.47 and 0.70), and for T_h = 9 μm (**m**, **n**; *P* = 0.28 and 0.119). Dotted red lines show 95th percentiles. **o**, **p** Actual clustering probabilities were plotted against simulated clustering probabilities (chance level) for generated spines (**o**) and eliminated spines (**p**). The small numbers next to the markers indicate threshold values. The contact point in (**o**) between the plot of VPA and the black dotted line parallel to the diagonal line provides Youden's index. See **Materials and Methods** for details. ***$P < 0.001$; *$P < 0.05$; n.s., not significant.

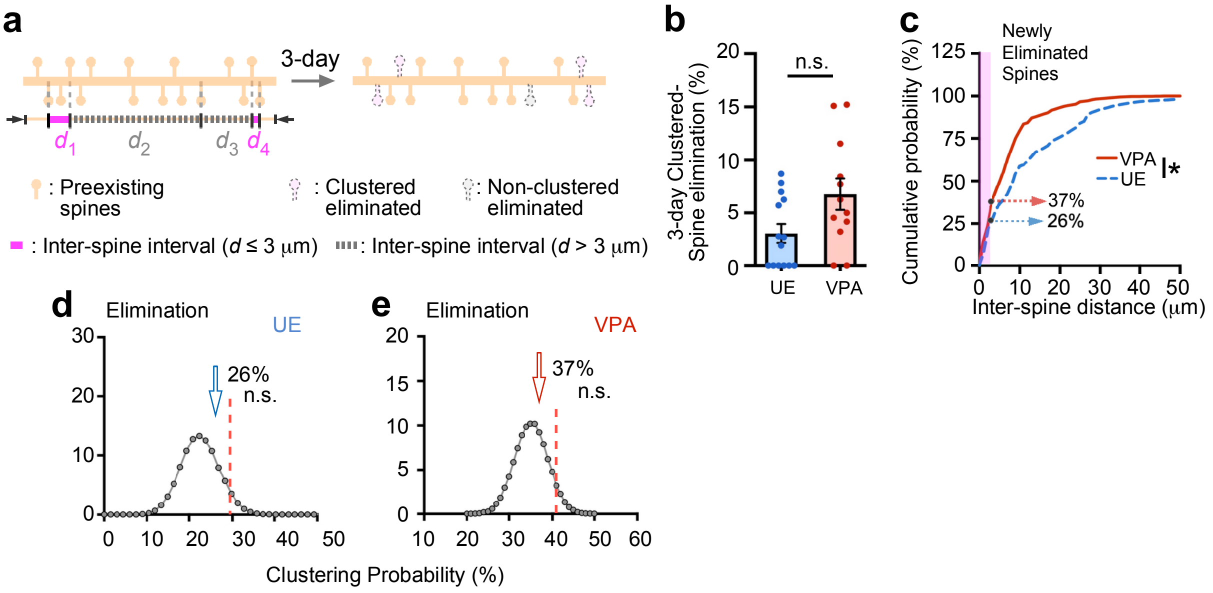

Supplementary Figure 3 The clustering bias of spine elimination was less pronounced than spine generation in VPA-exposed marmosets (related to Figure 2). a Schematic drawing of clustered spine elimination. Pairs of newly eliminated spines were considered to be clustered if they occurred within 3 μm of each other to detect proximal interaction. Spines colored pale-magenta or pale-gray represent clustered and non-clustered eliminated spines, respectively. **b** Comparison of the percentage of clustered eliminated spines to the total number of spines between UE and VPA-exposed animals (mean ± s.e.m.; $P = 0.065$, Mann-Whitney U test; $n = 14$ and 12 dendrites in four UE and three VPA-exposed animals, respectively). **c** Distribution of inter-spine distances between eliminated spines ($P = 0.016$; Kolmogorov-Smirnov test; $n = 67$ and 99 spine pairs in UE and VPA-exposed animals, respectively). Magenta-shaded area indicates inter-spine distances shorter than 3 μm, and numbers indicate the probabilities of clustering (within 3 μm). To prevent underestimation of the inter-spine distance, dendrites were concatenated into one long dendrite. **d**, **e** As in Fig. 2**e** and **f**, clustering bias of spine elimination was validated by Monte Carlo simulation. Circles connected with gray lines represent probability plots of clustering events from 100,000 simulations; the actual numbers of spine clusters are represented by arrows ($P = 0.20$ and 0.36; $n = 68$ and 100 newly eliminated spines in UE and VPA-exposed animals, respectively). Dotted red lines show 95th percentiles *$P < 0.05$; n.s., not significant.

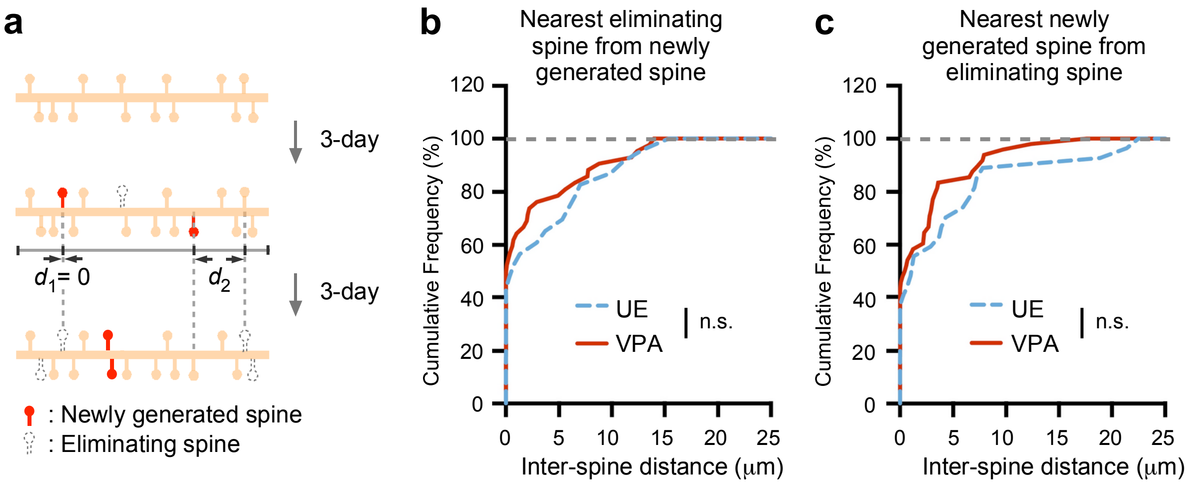

Supplementary Figure 4 Investigation of the heterogenous interaction between spine generation and spine elimination. **a** Schematic of the measurement of the distance between the newly generated spines and the eliminating spines to be removed in the next observation. The distance is zero if the generated spine itself is eliminated in the next session. **b** Cumulative frequency distribution of the distance from the newly generated spine to the nearest eliminating spine; no significant differences between the distributions for VPA-exposed and UE animals was observed (P = 0.77, Kolmogorov-Smirnov test; n = 23 and 42 generated-eliminating spine pairs for four UE and three VPA-exposed animals, respectively) . **c** Cumulative frequency distribution of the distance from the eliminating spine to the nearest newly generated spine. No significant differences between the distributions for VPA-exposed and UE animals was observed (P = 0.27, Kolmogorov-Smirnov test; n = 27 and 48 eliminating-generated spine pairs for four UE and three VPA-exposed animals). n.s., not significant.

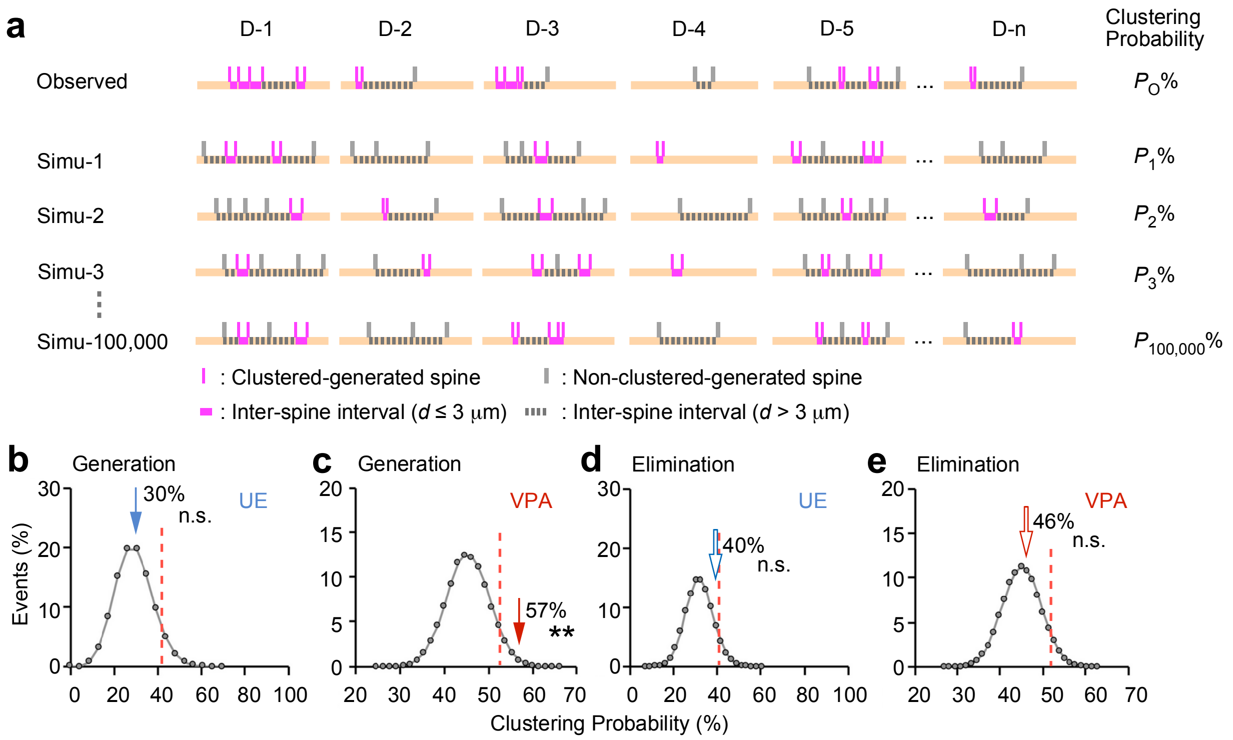

Supplementary Figure 5 Enhanced clustering of newly generated spines in VPA-exposed marmosets was further replicated when simulating their positioning within each dendrite (related to Figure 2). a Validation of spine clustering bias by another Monte Carlo simulation. In this simulation, the new spine positions were randomly determined with a uniform distribution without changing the spine number and dendrite length of each dendrite, since the new spine formation or elimination may occur in specific active dendrites. Clustering probabilities for all inter-spine distances were calculated for each simulation, and the distributions of the clustering probability from 100,000 iterations are shown in (**b**–**e**). **b**–**e** Circles connected with gray lines represent probability plots of clustering events from 100,000 simulations; the actual numbers of spine clusters are represented by arrows ($P = 0.33$ and 0.0062; $n = 23$ and 65 newly generated spine pairs in 14 and 12 dendrites in UE and VPA-exposed animals, respectively) ($P = 0.083$ and 0.35; $n = 43$ and 78 newly eliminated spine pairs in 14 and 12 dendrites in UE and VPA-exposed animals, respectively). Dotted red lines show 95th percentiles. See **Materials and Methods** for details. $^{**}P < 0.01$; n.s., not significant.

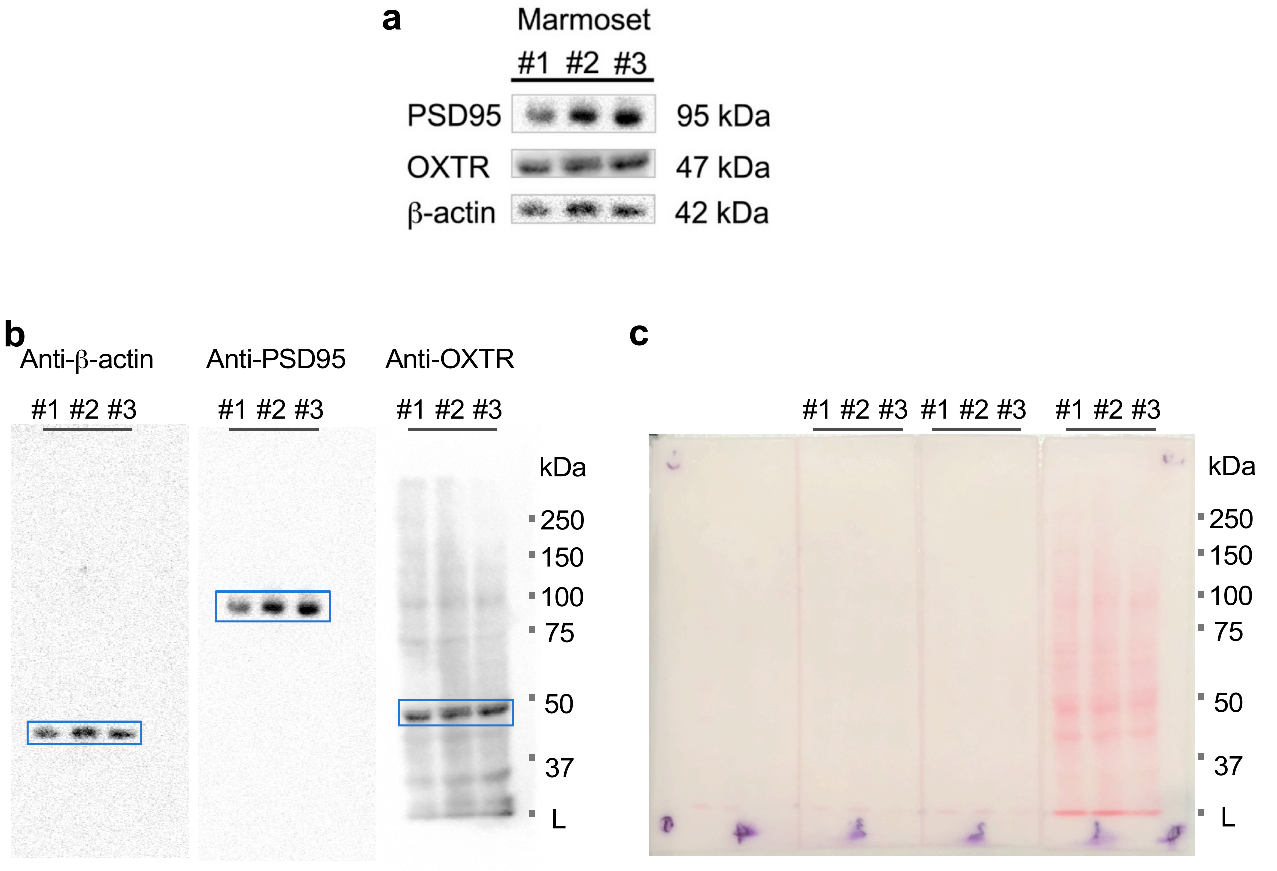

Supplementary Figure 6 Oxytocin receptors are detected in the synaptic fraction of the marmoset's prefrontal cortex gray matter. **a** Oxytocin receptors (OXTR) along with post-synaptic protein PSD95 and β-actin are detected using western blotting in the synaptosomal fraction derived from the prefrontal cortex gray matter of three adult marmosets. **b** Unedited western blots. **c** Ponceau S stained PVDF membranes for the blots.

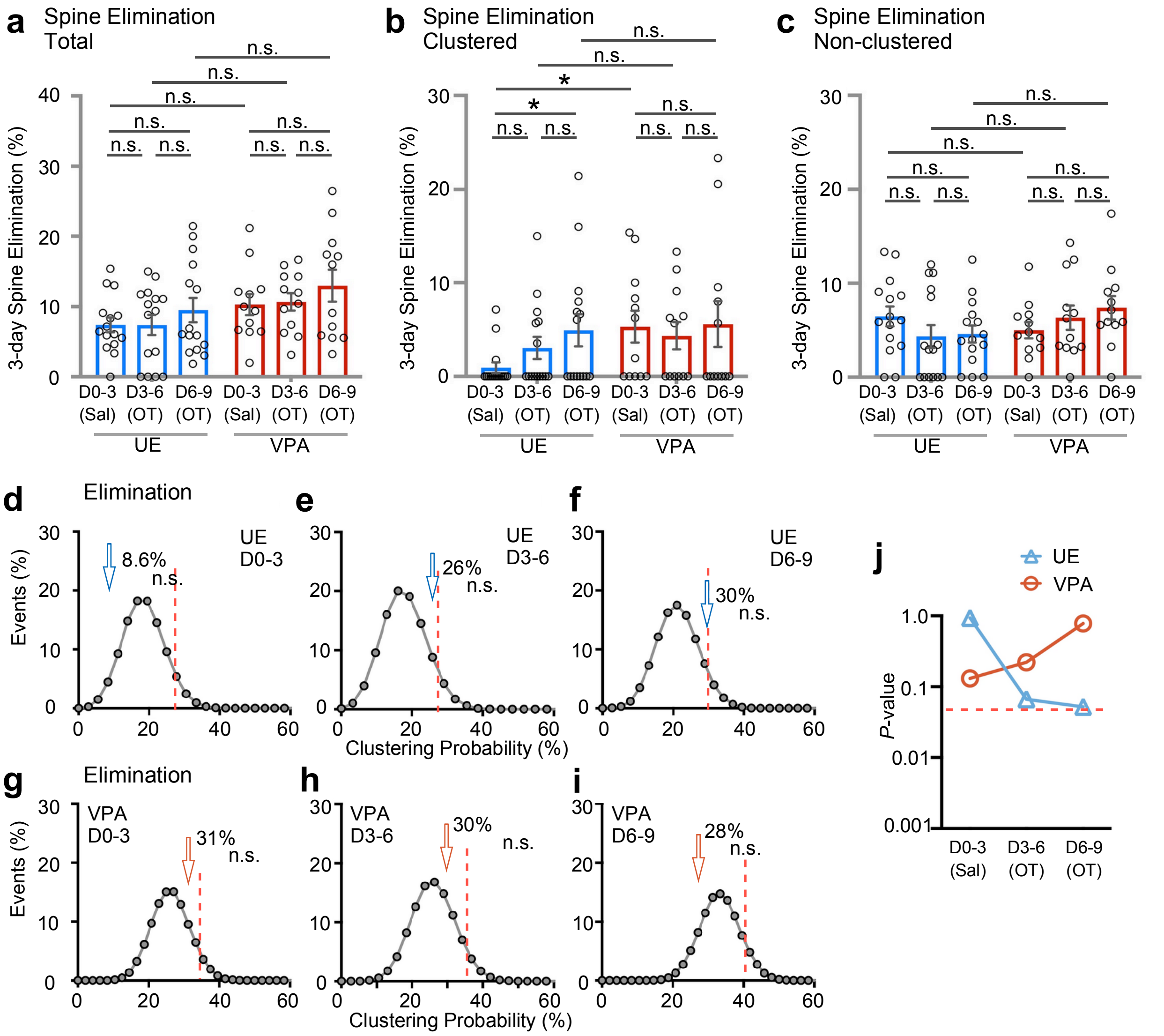

Supplementary Figure 7 Effect of oxytocin on spine elimination proximity in UE and VPA-exposed marmosets. **a** Three-day total spine elimination rates (mean ± s.e.m.; Two-way ANOVA, group: $P = 0.044$, time: $P = 0.18$, interaction: $P = 0.98$; *post hoc* Tukey's test, D0–3 (UE vs. VPA): $P = 0.14$; $n = 15$ dendrites from four UE animals; $n = 12$ dendrites from three VPA-exposed animals). **b** Three-day clustered spine elimination rates (mean ± s.e.m.; Two-way ANOVA, group: $P = 0.14$, time: $P = 0.33$, interaction: $P = 0.41$; *post hoc* Tukey's test, D0–3 (UE vs. VPA): $P = 0.028$, UE (D0–3 vs. D6–9): $P = 0.036$; $n = 15$ dendrites from four UE animals; $n = 12$ dendrites from three VPA-exposed animals). **c** Three-day non-clustered spine elimination rates (mean ± s.e.m.; Two-way ANOVA, group: $P = 0.25$, time: $P = 0.82$, interaction: $P = 0.13$; *post hoc* Tukey's test, D0–3 (UE vs. VPA): $P = 0.28$; $n = 15$ dendrites from four UE animals; $n = 12$ dendrites from three VPA-exposed animals). **d–i** Simulation analysis to analyze the effects of oxytocin on clustering bias of newly eliminated spines in the UE (**d**–**f**) and VPA-exposed (**g**–**i**) animals ($n = 21$, 21, and 25 newly eliminated spine pairs from four UE animals, and $n = 34$, 27, and 37 pairs from three VPA-exposed animals, during the D0–3, D3–6, and D6–9 periods, respectively). Dotted red lines show 95th percentiles. **j** The P-values expressed in logarithm from (**d**–**i**) are indicated. *$P < 0.05$; n.s., not significant.

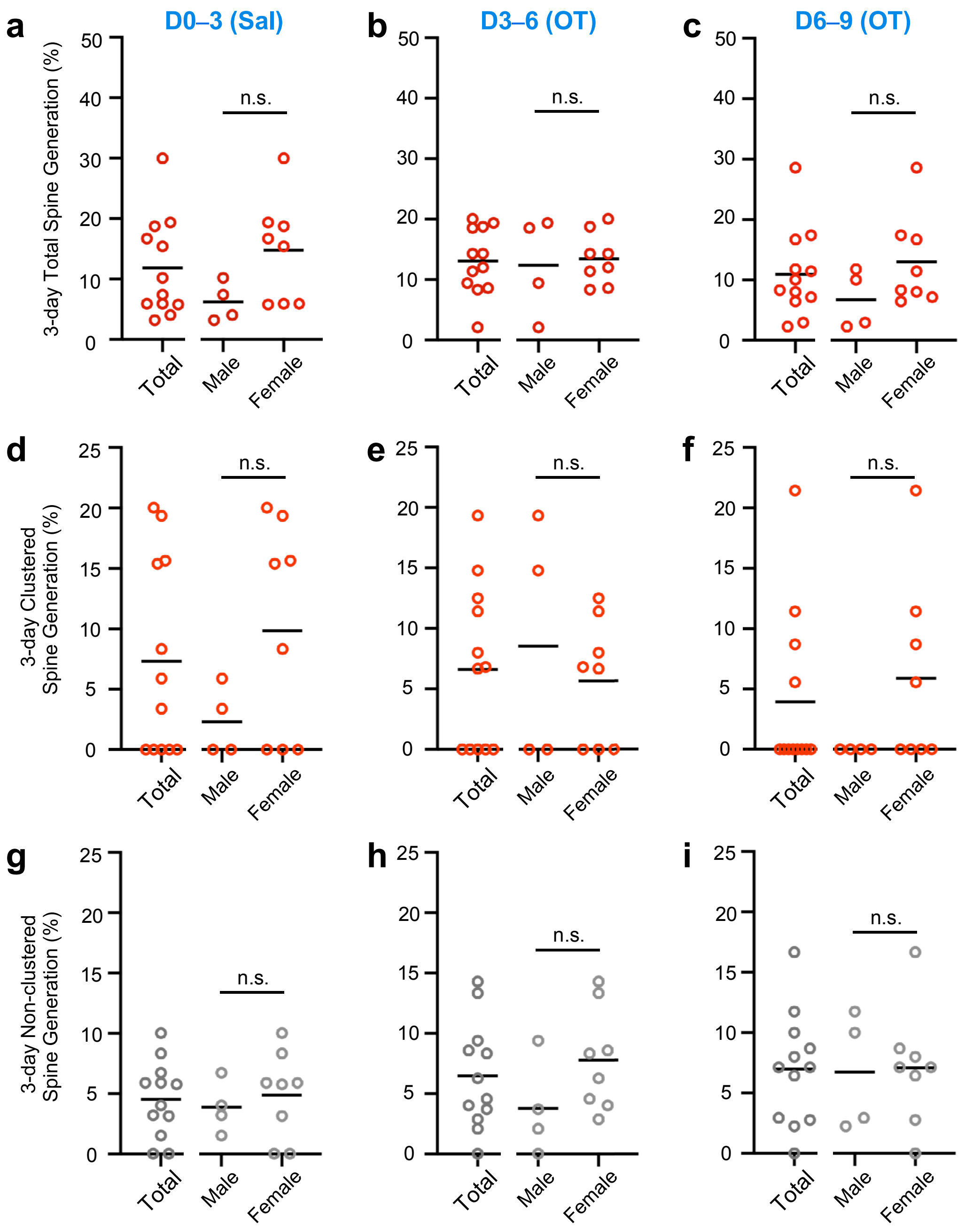

Supplementary Figure 8 Investigation of sex differences in modifying spine generation subsequent to oxytocin administration to VPA-exposed marmosets (Related to Fig. 5). Each dendrite is depicted through scatter plots to assess sexual dimorphism in spine dynamics. The total and sex-specific generation rates are delineated separately. Horizontal bars indicate average values. Subsections (**a**–**c**), (**d**–**f**), and (**g**–**i**) present the three-day spine generation rates, clustered spine generation rates, and non-clustered spine generation rates, respectively. Statistical analysis using the Mann-Whitney test indicates no significant differences between male and female groups across all time intervals (**a**–**c**, $P = 0.099$, 0.90 and 0.28; **d**–**f**, $P = 0.26$, 0.67 and 0.21; **g**–**i**, $P = 0.78$, 0.15 and 0.91, for D0–3, D3–6 and D6–9, respectively; $n = 12$ dendrites in three VPA-exposed animals). n.s., not significant.

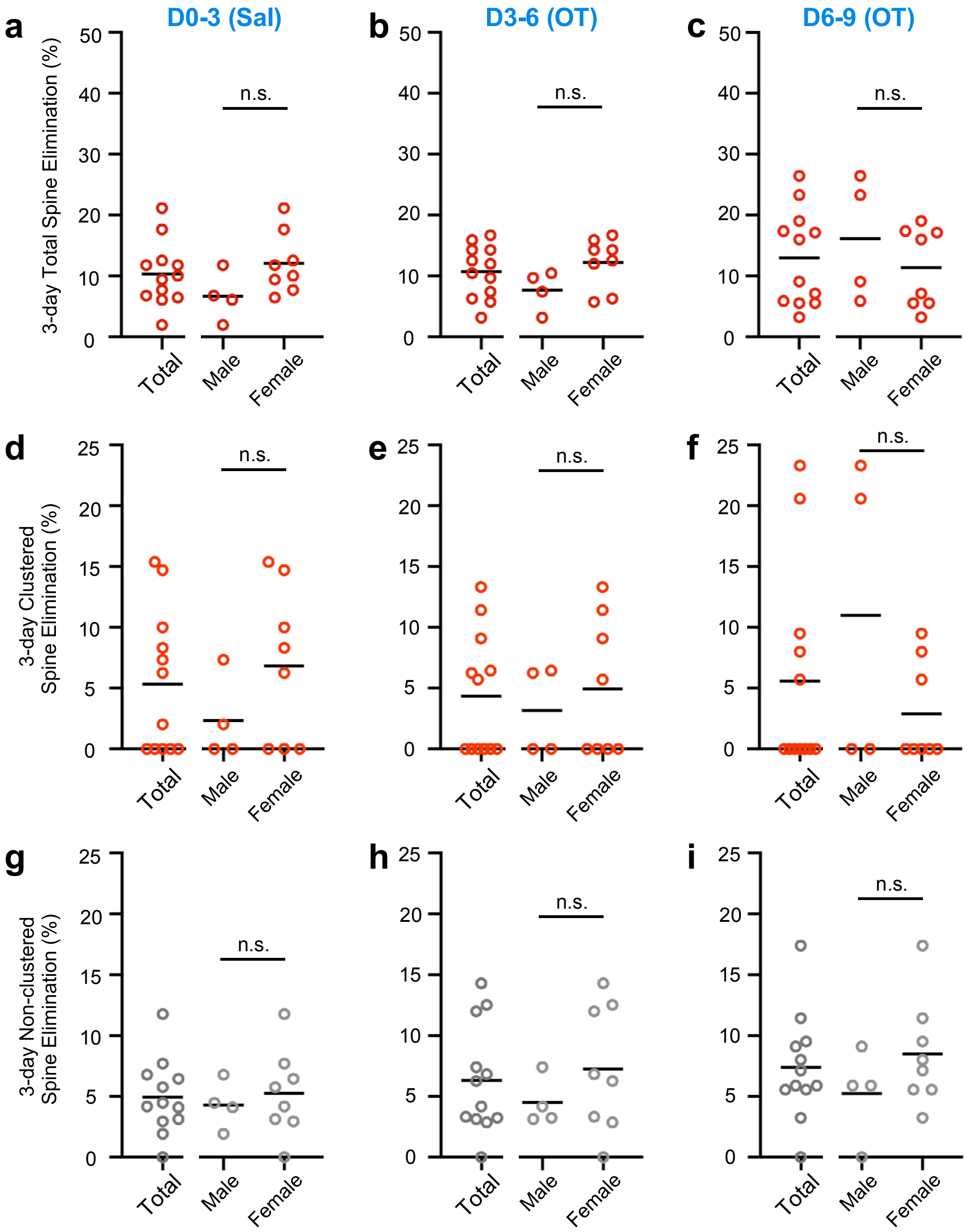

Supplementary Figure 9 Investigation of sex differences in modifying spine elimination subsequent to oxytocin administration to VPA-exposed marmosets (Related to Supplementary Fig. 7). Each dendrite is depicted through scatter plots to assess sexual dimorphism in spine dynamics. The total and sex-specific elimination rates are delineated separately. Horizontal bars indicate average values. Subsections (**a**–**c**), (**d**–**f**), and (**g**–**i**) present the three-day spine elimination rates, clustered spine elimination rates, and non-clustered spine elimination rates, respectively. Statistical analysis using the Mann-Whitney test indicates no significant differences between male and female groups across all time intervals (**a**–**c**, $P = 0.11$, 0.26 and 0.10; **d**–**f**, $P = 0.78$, 0.59 and 0.41; **g**–**i**, $P = 0.81$, 0.57 and 0.44, for D0–3, D3–6 and D6–9, respectively; $n = 12$ dendrites in three VPA-exposed animals). n.s., not significant.

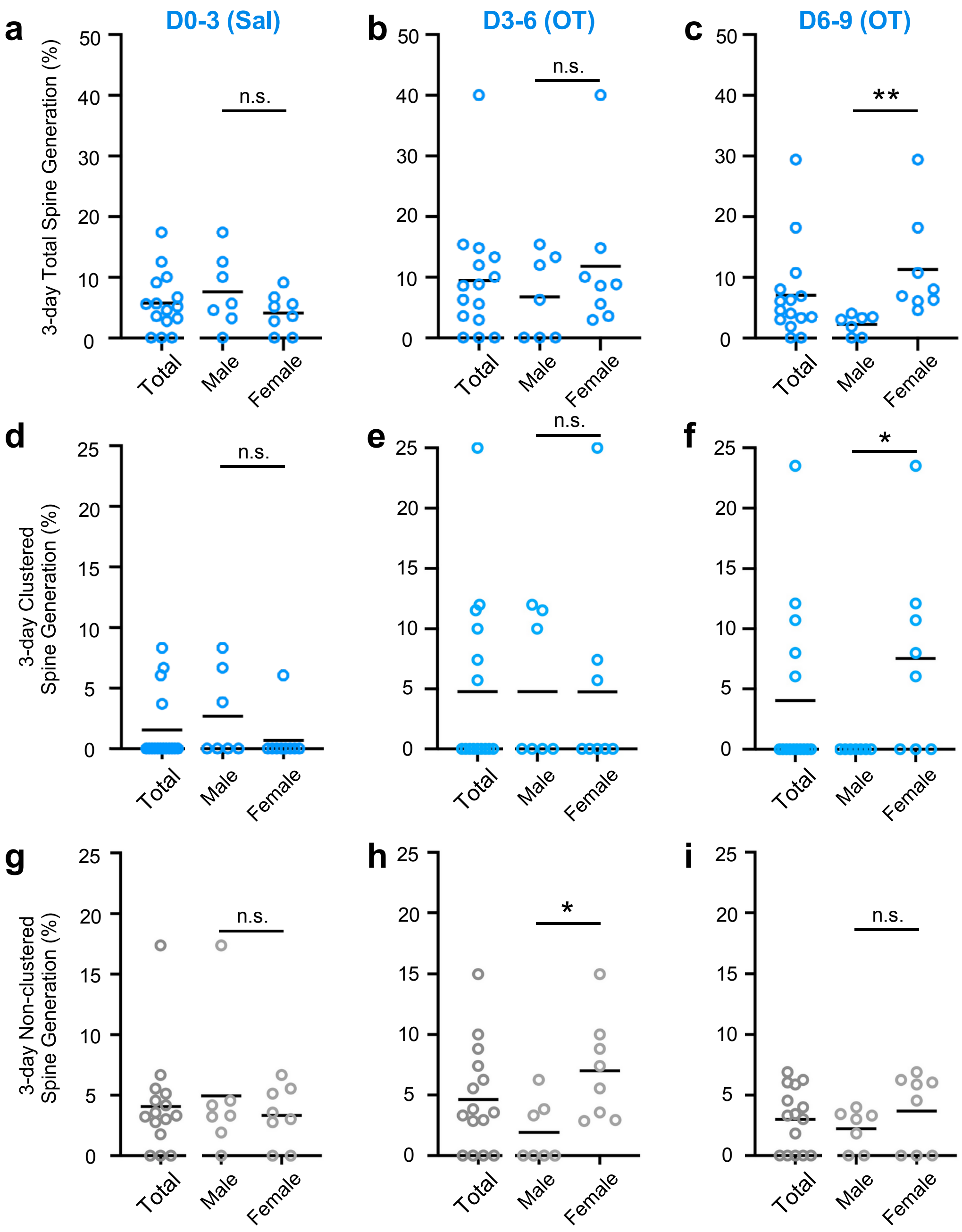

Supplementary Figure 10 Investigation of sex differences in modifying spine generation subsequent to oxytocin administration to UE marmosets (Related to Fig. 5). Each dendrite is depicted through scatter plots to assess sexual dimorphism in spine dynamics. The total and sex-specific generation rates are delineated separately. Horizontal bars indicate average values. Subsections (**a**–**c**), (**d**–**f**), and (**g**–**i**) present the three-day spine generation rates, clustered spine generation rates, and non-clustered spine generation rates, respectively. Statistical analysis using the Mann-Whitney test indicates significant differences between male and female groups only in the total spine generation and the clustered spine generation at D6–9 condition (**a**–**c**, $P = 0.28$, 0.53 and 0.003; **d**–**f**, $P = 0.18$, 0.75 and 0.026; **g**–**i**, $P = 0.96$, 0.026 and 0.27, for D0–3, D3–6 and D6–9, respectively; $n = 15$ dendrites in four UE animals). $^{**}P < 0.01$; $^{*}P < 0.05$; n.s., not significant.

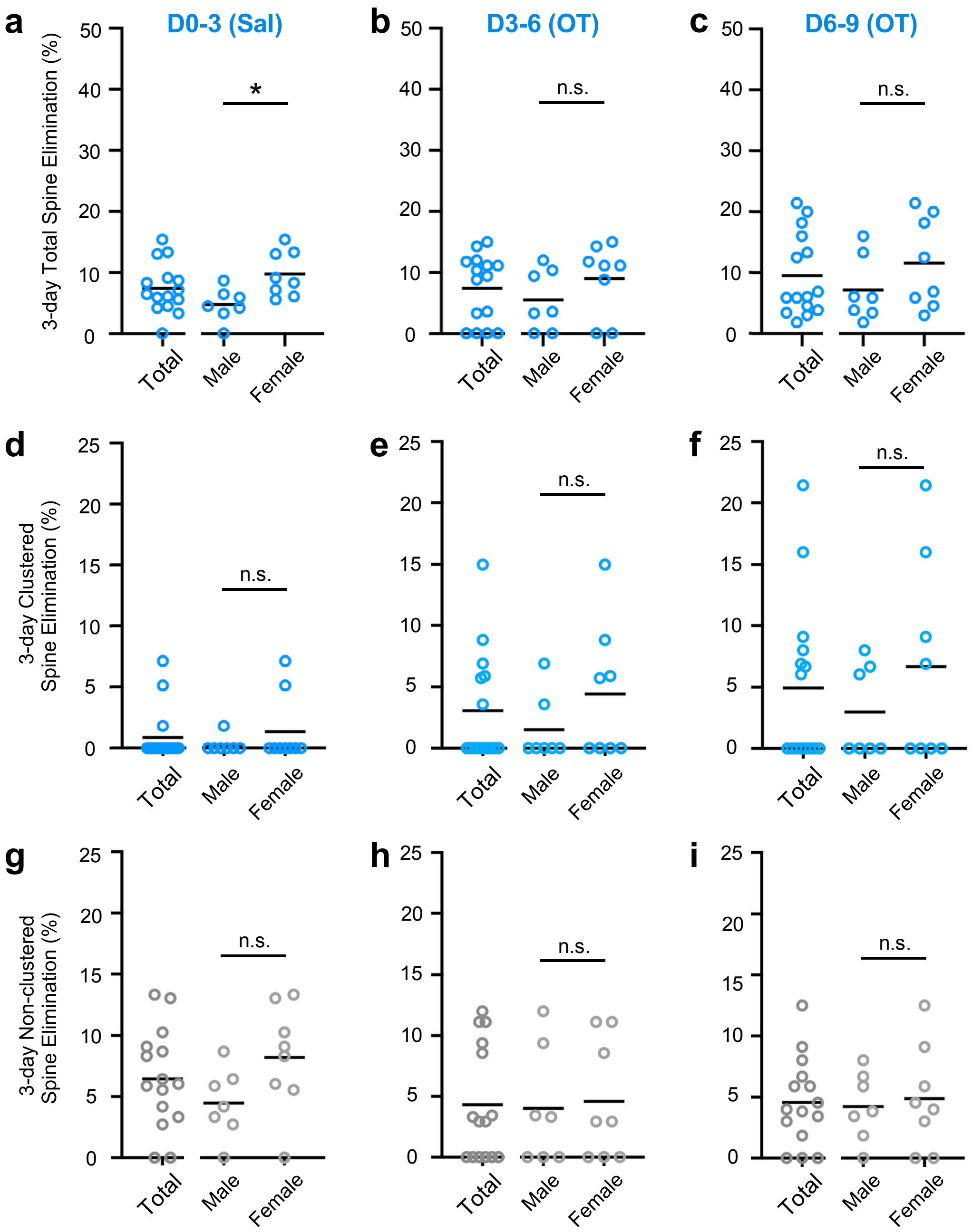

Supplementary Figure 11 Investigation of sex differences in modifying spine elimination subsequent to oxytocin administration to UE marmosets (Related to Supplementary Fig. 7). Each dendrite is depicted through scatter plots to assess sexual dimorphism in spine dynamics. The total and sex-specific elimination rates are delineated separately. Horizontal bars indicate average values. Subsections (**a**–**c**), (**d**–**f**), and (**g**–**i**) present the three-day spine elimination rates, clustered spine elimination rates, and non-clustered spine elimination rates, respectively. Statistical analysis using the Mann-Whitney test indicates significant differences between male and female groups only in the total spine elimination at D0–3 condition (**a**–**c**, $P = 0.014$, 0.26 and 0.24; **d**–**f**, $P = 0.34$, 0.62 and 0.40; **g**–**i**, $P = 0.075$, 0.98 and 0.89, for D0–3, D3–6 and D6–9, respectively; $n = 15$ dendrites in four UE animals). *$P < 0.05$; n.s., not significant.

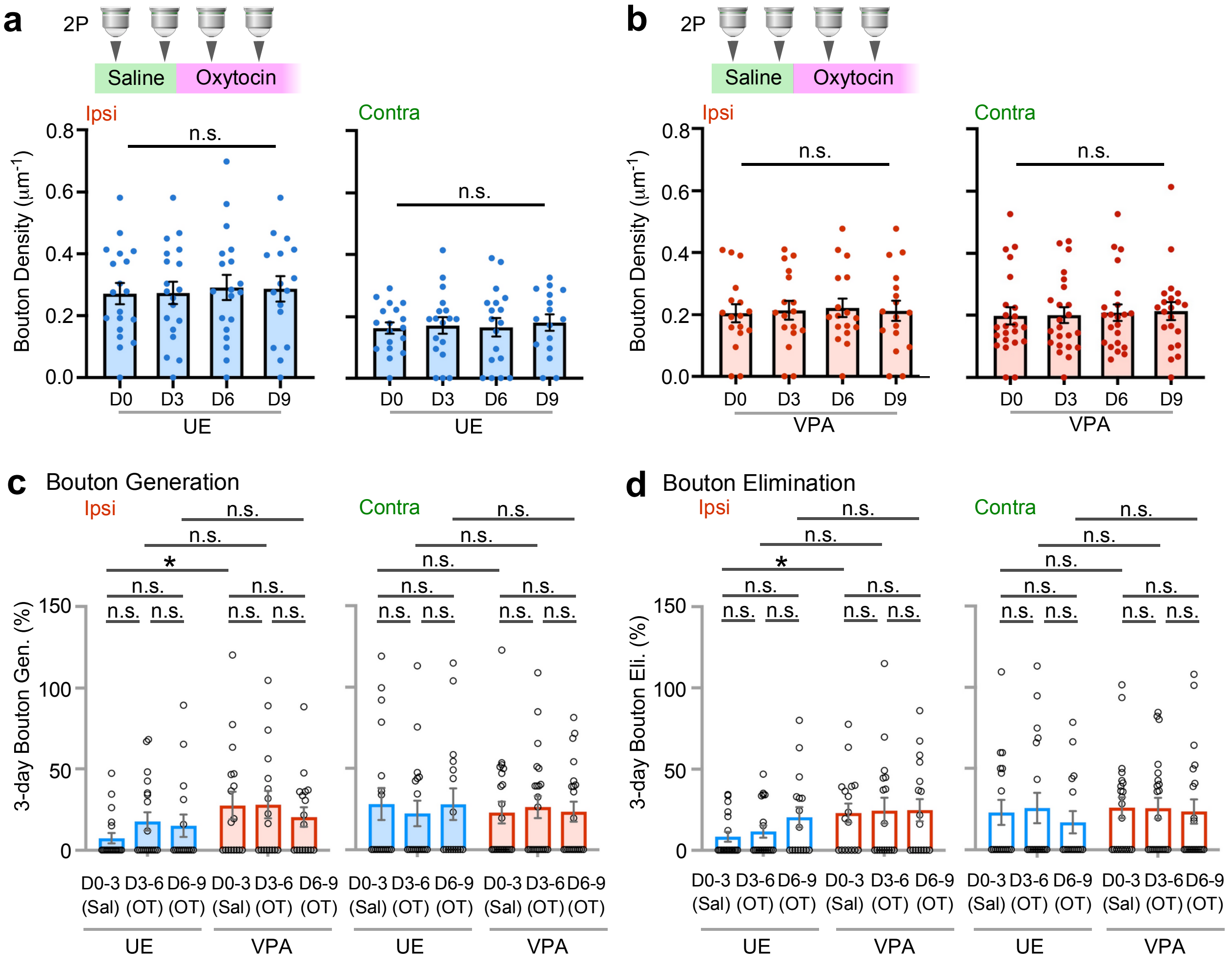

Supplementary Figure 12 Regulation of axonal bouton generation and elimination by oxytocin. **a**, **b** Mean bouton density values of UE (**a**) and VPA-exposed (**b**) marmoset axons before and after oxytocin administration. The bouton densities were separately displayed for ipsilateral (Left panel) and contralateral axons (Right panel) (mean ± s.e.m.; P = 0.98, 0.92, 0.997, and 0.95; n = 19, 18, 17 and 22 axons for UE-ipsi, UE-contra, VPA-ipsi, and VPA-contra, respectively; Kruskal-Wallis test). **c** Three-day bouton generation during the D0–3, D3–6, and D6–9 periods for ipsilateral and contralateral axons (ipsi: mean ± s.e.m.; Two-way ANOVA, group: P = 0.038, time: P = 0.64, interaction: P = 0.51; *post hoc* Tukey's test, D0–3 (UE vs. VPA): P = 0.038; n = 19 axons from three UE animals; n = 17 axons from two VPA-exposed animals)(contra: mean ± s.e.m.; Two-way ANOVA, group: P = 0.75, time: P = 0.98, interaction: P = 0.80; *post hoc* Tukey's test, D0–3 (UE vs. VPA): P = 0.66; n = 18 axons from three UE animals; n = 22 axons from two VPA-exposed animals). **d** Three-day bouton elimination displayed as in (**c**) (ipsi: mean ± s.e.m.; Two-way ANOVA, group: P = 0.050, time: P = 0.45, interaction: P = 0.61; *post hoc* Tukey's test, D0–3 (UE vs. VPA): P = 0.036; n = 19 axons from three UE animals; n = 17 from two VPA-exposed animals)(contra: mean ± s.e.m.; Two-way ANOVA, group: P = 0.61, time: P = 0.74, interaction: P = 0.90; *post hoc* Tukey's test, D0–3 (UE vs. VPA): P = 0.78; n = 18 axons from three UE animals; n = 22 axons from two VPA-exposed animals). In the Two-way ANOVA in (**c**) and (**d**), a mixed-effects model was used to complement missing values. *P < 0.05; n.s., not significant.
