# Supplementary material for: Altered projection-specific synaptic remodeling and its modification by oxytocin in an idiopathic autism marmoset model
Source: Commun Biol. 2024 May 27;7:642. doi: 10.1038/s42003-024-06345-9 (PMC11130163; doi:10.1038/s42003-024-06345-9)
Supplement: Supplementary file 3 — Description of Additional Supplementary Files [file 42003_2024_6345_MOESM3_ESM.pdf]

Description of Additional Supplementary Files

File name: Supplementary Data 1

Description: List of examined genes in gene chip analysis of VPA-exposed marmosets and expression of corresponding genes from human ASD data (Parikshak et al. [2016]).

File name: Supplementary Data 2

Description: A gene pathway analysis performed using IPA software for each cell type based on gene expression.

File name: Supplementary Data 3

Description: The source data behind the graphs in the paper.
